# Errors in Imagined and Executed Typing

**DOI:** 10.3390/vision3040066

**Published:** 2019-11-20

**Authors:** Stephan F. Dahm, Martina Rieger

**Affiliations:** Department of Psychology and Medical Sciences; Institute of Psychology, UMIT–Private University for Health Sciences, Medical Informatics and Technology, 6060 Hall in Tyrol, Austria; martina.rieger@umit.at

**Keywords:** motor imagery, typing style, feedback, internal monitoring, forward models

## Abstract

In motor imagery (MI), internal models may predict the action effects. A mismatch between predicted and intended action effects may result in error detection. To compare error detection in MI and motor execution (ME), ten-finger typists and hunt-and-peck typists performed a copy-typing task. Visibility of the screen and visibility of the keyboard were manipulated. Participants reported what type of error occurred and by which sources they detected the error. With covered screen, fewer errors were reported, showing the importance of distal action effects for error detection. With covered screen, the number of reported higher-order planning errors did not significantly differ between MI and ME. However, the number of reported motor command errors was lower in MI than in ME. Hence, only errors that occur in advance to internal modeling are equally observed in MI and ME. MI may require more attention than ME, leaving fewer resources to monitor motor command errors in MI. In comparison to hunt-and-peck typists, ten-finger typists detected more higher-order planning errors by kinesthesis/touch and fewer motor command errors by vision of the keyboard. The use of sources for error detection did not significantly differ between MI and ME, indicating similar mechanisms.

## 1. Introduction

Motor imagery (MI) consists of a mental rehearsal of one’s own action without actually performing the action [1]. In the applied setting, MI is used in mental practice and in brain computer interfaces [2,3,4]. Due to those applications, it is important to understand the underlying mechanisms of MI. In many respects, MI seems to rely on similar mechanisms as motor execution (ME) [1,5,6,7,8,9,10,11,12]. However, there are also differences between MI and ME [11,12,13,14,15,16]. One such difference is that MI requires not only the imagination of the action, but also the imagination of the action effects on the body and the environment. These action effects may include errors. The question arises whether and how errors are detected in MI. In the present study, we used a typing task to investigate the online detection of error occurrence in imagined and executed typing. Typing is particularly suited to investigate errors, because errors are easy to observe and occur frequently. In particular, we investigated how the lack of actual feedback contributes to differences between MI and ME in ten-finger typists and hunt-and-peck typists. Further, we investigated whether differences between MI and ME occur similarly for higher-order planning errors and motor command errors. In addition, we were interested in which sources of feedback are used for error detection in MI and ME.

According to the comparator model of motor control, in ME errors can be detected by comparing intended action effects, internally predicted action effects, and observed action effects [17,18]. A movement starts with the intention to reach a certain goal (intended action effects). Then, an inverse model selects the corresponding motor commands, which contain information about sequencing, relative force, and relative timing of the movement [19]. When the movement is executed, actual action effects occur, which can be observed. An internal prediction of action effects is achieved by forward models that use an efference copy of the motor commands to compute the outcomes of the motor commands [17,18]. A comparison of intended action effects and predicted action effects results in an error signal when there is a mismatch. This enables the detection of potential action errors even before an action is executed [20], and can modulate the motor command [21].

**Figure 1:**
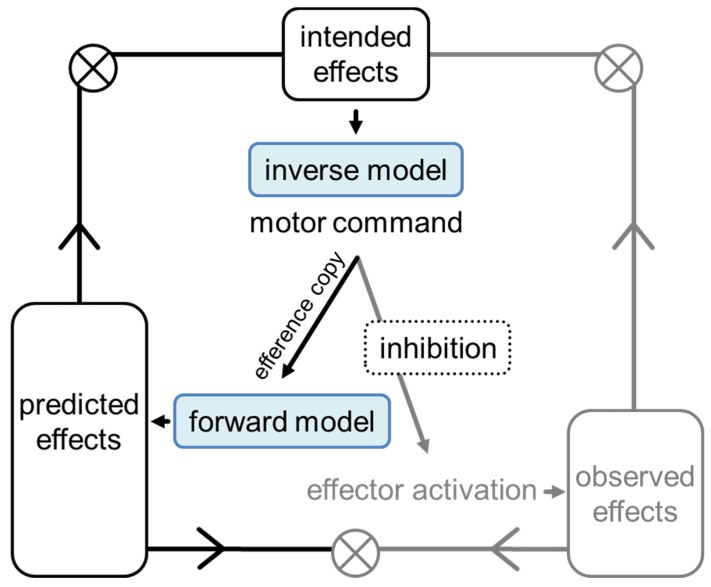
Framework of internal models (adapted from [26]). The mechanisms in black may be used in both motor execution and motor imagery. The mechanisms in gray may not be used (or to a lesser degree) in motor imagery due to inhibition.

In MI, assuming that motor commands are generated, forward models may predict the action effects in a similar way [22,23] (Figure 1). However, in MI, the comparison of observed and predicted action effects and the comparison of observed and intended effects cannot occur, because no actual action effects are available. Only the comparison of predicted and intended action effects can occur. For the experience of errors during MI, the occurrence of error signals due to this comparison must be internally monitored. Of central interest in the present study are the questions whether internal predictions and whether internal monitoring take place in MI. It has been suggested that in MI, motor commands are sometimes inhibited before an efference copy is made [24], predictions are imprecise, error monitoring is neglected, or attention to automatic processes requires many resources such that less are available for error monitoring [25]. These suggestions are based on the observation that differences between MI and ME cannot be explained solely by the lack of distal feedback from the environment. For instance, fewer typing errors related to execution processes are reported in MI than in ME, even when the screen is covered in ME [25].

In the present study, we used a copy-typing task to investigate the online detection of error occurrence in imagined and executed typing. We intended to replicate and extend previous findings [25]. It has previously been shown that in typing, most errors are reported in execution with visible screen, fewer errors are reported in execution with covered screen, and fewest errors are reported in imagination [25]. However, in that study, participants were asked to report errors after they completed typing a whole proverb. This might lead to biases in reporting, as the occurrence of some errors might have been forgotten by the end of a proverb. In addition, because participants received relatively short text templates, overall very few errors occurred, even in ME. Therefore, in the present study, we used longer text templates to increase the number of errors. Further, participants were asked to stop typing immediately when they noticed the occurrence of an error and to report the error. By this, we aimed to investigate immediate error detection in both ME and MI.

Again, participants performed execution with visible screen, execution with covered screen, and imagination to control for the role of visual feedback from the screen on differences between imagination and execution [25]. In addition, we investigated the effects of covering the keyboard or not, because even expert typists occasionally look at the keyboard to find rarely used keys [27]. Thus, we expected more errors when the keyboard is covered than when it is visible, and, assuming similar mechanisms in MI and ME, we expected that the influence of keyboard visibility does not significantly differ between ME and MI.

In copy typing, different types of errors occur. Higher-order planning errors (i.e., word omissions) occur because the intended action does not coincide with the requested action. Higher-order planning errors include errors in which one does not plan to perform an action or one plans to perform an action that is not requested (e.g., one may plan to type something that is not written on the template). Such errors result from incorrectly specified intentions. In contrast, in motor command errors, one does intend to type what is written on the template, but the internal model is incorrectly specified. For instance, the specification of the finger, the hand, the movement direction [28], or the timing of consecutive keypresses [29] may be incorrect. Previous results indicate that higher-order planning errors are equally often reported in MI and ME, whereas motor command errors are less often reported in MI than in ME [25]. We expected to replicate those findings [25]. Still, we expected that at least some motor command errors are reported in MI [25]. Reports of some motor command errors in MI would indicate that internal predictions and internal monitoring take place in MI.

Different sources of feedback can contribute to error detection in typing. In particular, visual feedback from the screen is an important source for error detection [25,27,30,31]. Distal action effects from the screen usually provide definite knowledge about the actual action effects. Feedback from the screen is so important, that typists even perceive false feedback on the screen as an effect of their own action [31]. However, further sources of feedback contribute to error detection as well: visual feedback from the keyboard, visual feedback from the fingers, tactile feedback from touching the keys, kinesthetic feedback from the movement of the fingers, and internal monitoring. Further, errors may be detected by reviewing the planning process—for instance, by reviewing one’s own intentions. Therefore, in the present study, we investigated how participants detect typing errors in execution and imagination. We asked participants to describe how they detected each reported error. In MI and ME without actual feedback, we expected that participants spontaneously imagine external events (for instance, visual information from the screen and keyboard) and use this information to detect errors [32]. Further, we did not expect significant differences between MI and ME in the use of error detection sources that do not depend on feedback (internal monitoring, reviewing one’s own intention).

We further investigated typists using different typing styles. Ten-finger typists use multiple fingers and have a fixed finger to key assignment, e.g., they always press the “s” key with the left ring finger. In ten-finger typists, effector-dependent representations are automatically activated when seeing a letter [33]. They rarely look at the keyboard and their fingers. This allows them to pay more attention to the screen or to the template (in copy typing). In contrast, hunt-and-peck typists use fewer fingers, and specific letters are not automatically associated with a specific finger movement. Hunt-and-peck typists often have to look at the keyboard to find the exact position of the required key. Correspondingly, ten-finger typists report that they use kinesthesis/touch and internal monitoring more often in order to detect an error than idiosyncratic typists [34]. Therefore, we expected to find that error detection in ten-finger typists relies relatively more on kinesthetic/tactile feedback and the screen, whereas error detection in hunt-and-peck typists relies relatively more on visual feedback from the keyboard and fingers [34]. We further expected that ten-finger typists differ less in the use of sources for error detection in MI and ME than hunt-and-peck typists, because ten-finger typists presumably have more precise internal models of typing [14]. Further, due to more precise internal models, predictions of action effects should be more precise in ten-finger typists than in hunt-and-peck typists [14]. Thus, in MI, we expected more reported motor command errors in ten-finger typists than in hunt-and-peck typists.

## 2. Methods

### 2.1. Participants

Originally, the study involved 31 ten-finger typists and 31 hunt-and-peck typists. Ten-finger typists used at least nine fingers (four ten-finger typists were using only one thumb) and were able to type without looking at the keyboard. Hunt-and-peck typists used a maximum of two fingers per hand. Participants who stopped reporting errors in the course of an experimental session to shorten the duration of data collection were excluded from the analysis. Demographic data, data on typing experience, and the results of a copy-typing test (consisting of 94 words/705 keystrokes) of the remaining ten-finger typists (*N* = 25) and hunt-and-peck typists (*N* = 29) are shown in Table 1. Participants were paid nine Euros per hour or received course credit. All participants gave informed consent.

### 2.2. Material

**Figure 2:**
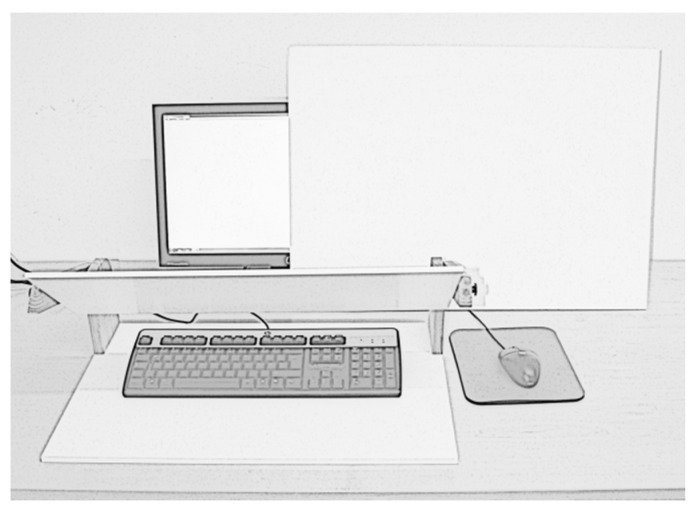
Illustration of the experimental setup. To manipulate visual feedback from the fingers and the keyboard, a wooden board was adjusted so that the fingers and the keyboard were either visible (as depicted) or not. To manipulate visual feedback from the screen, a piece of cardboard was placed either beside the screen or in front of the screen.

Participants typed on a computer with a standard German keyboard (QWERTZ). Three texts (653, 922, and 1166 keystrokes) from a magazine for learning German as a foreign language [35,36,37], in which we eliminated or replaced characters that are not typed very often (exclamation marks, semicolons and parentheses), served as templates for copy typing. The templates were printed in black on a white sheet of paper (font Calibri, font size 11, double spaced) and were placed at the left side of the keyboard on the table. We chose this setup rather than presenting the template on the screen because we consider this an ecologically valid situation. For instance, a student may copy type important content from a textbook this way. With a digital template a student may use the copy and paste function on the computer rather than perform copy typing. An adjustable wooden board was used to manipulate the visibility of the fingers and the keyboard. A white piece of cardboard was used to manipulate the visibility of the screen (see Figure 2). The KBLog program [38] was used to register every keystroke during the whole experiment.

### 2.3. Design and Procedure

All participants were tested individually in three sessions. In each session, a different text served as a template. In the beginning of the first session, participants performed a short typing test. Then, to familiarize participants with the experimental conditions, participants performed all conditions typing a short sentence. After that, data collection started.

In each session, participants performed all experimental conditions. They were asked to read the template before they started each experimental condition. Participants were asked to execute typing with feedback from the screen (EXE+S), execute typing without visual feedback from the screen (EXE-S), and imagine typing (IMA). In the *execution* conditions, participants started with their fingers on the keyboard. In the *imagination* conditions, participants placed their fingers in front of the keyboard on the table. Participants were asked to keep the fingers motionless and to imagine how it feels to perform the movements. Ten-finger typists performed all conditions once with visible keyboard and once with covered keyboard. Hunt-and-peck typists only performed the conditions with visible keyboard, because preliminary testing showed that with covered keyboard, hunt-and-peck typists were searching for keys and were trying to acquire a representation of the QWERTZ keyboard. They were unsure whether they pressed the correct key in each keystroke. Thus, ten-finger typists performed six conditions, and hunt-and-peck typists performed three conditions. The order of conditions was counter-balanced across participants.

Participants were asked to stop (their imagination or execution of) typing when they noticed committing a typing error. Participants were told that every wrong key press counted as an error, regardless whether the text on the screen was correct or incorrect (e.g., pressing the shift key and releasing it again before typing a letter in lowercase does not result in an error on the screen). Participants were asked to indicate the error’s position in the text and to report what they had typed. They were further asked to state how they had noticed the error. Participants were told that errors in typing can be detected in different ways: by realizing that the template had been read incorrectly, by noticing that a wrong key had been pressed, by looking at the keyboard, by looking at the screen, by noticing that something is going wrong without using any particular source of information, or by other reasons. To illustrate this procedure, participants may have written “Oktobervest” instead of “Oktoberfest”. They stopped and mentioned that they wrote a “v” instead of an “f” in the word Oktoberfest and that they had noticed the error by looking at the screen. Hereafter, they continued typing.

Error correction was explicitly not required. Participants were asked to type as fast as possible in all conditions. Time pressure was intended to induce a high number of errors. Participants were asked not to commit errors intentionally, but to report every error that occurred. An experimenter recorded the reports.

### 2.4. Data Analysis

After the experiment, a second experimenter categorized errors into subcategories [28,29] based on participants’ reports on where the error occurred and what they had typed. For a complete overview with examples of all the subcategories, see Appendix A. In case the assignment was not clear, a third experimenter was consulted. Then, the subcategories were combined into higher-order planning errors (e.g., substitutions of a word), motor command errors (e.g., insertions of a neighboring key), and subcategories that were not assignable to either of these categories. The latter were not included in the following analyses.

Further, participants’ reports of how they detected errors were assigned to different sources for error detection. The five resulting categories were named as follows: reviewing the planning process, vision of the screen, vision of the keyboard, kinesthesis/touch, and internal monitoring (Appendix B). The category reviewing the planning process was assigned when participants reported that they made an orthographic mistake, that they misread the template, or that they forgot to type a letter. The category kinesthesis/touch was assigned when participants reported that they detected the error because they felt that they made wrong keystrokes, they felt they used the wrong finger, or they felt they made a wrong movement. We did not differentiate between kinesthesis and touch, because preliminary testing has shown that participants sometimes have difficulties differentiating between them in typing. The category internal monitoring was assigned when participants had the impression that something was going wrong, but could not name a specific source for error detection.

The number of reported errors was analyzed using repeated measures analyses of variance (ANOVAs). False alarms and incorrectly identified errors were included in the number of reported errors, because they cannot be identified in imagination. If Mauchly’s test indicated that the assumption of sphericity was violated, we report Huyn–Feld corrected degrees of freedom and *p*-values. Post hoc comparisons were conducted using t-tests with Sidak adjusted pairwise comparisons. Where appropriate, we report means, standard errors (±), minimum (*p*_min_, *d*_min_), and maximum (*p*_max_*, d*_max_) statistical values. Statistical significance was set at *p* < 0.05. Data are provided here: https://doi.org/10.17605/OSF.IO/82TG3. Further, a report of additional analyses, beyond those reported in the paper, is provided in the Supplement. Additional analyses were performed on the number of actual errors, the hit rate, the false alarm rate, the percentage of actual errors of reported errors, the percentage of incorrectly identified errors (participants correctly reported that an error occurred, but were not correct about what had actually gone wrong), and the position of reported errors within words.

## 3. Results

Means and standard errors of the number of reported errors are shown in Table 2.

### 3.1. Ten-Finger Typists: Keyboard, Action, Error Type, and Source

An ANOVA with the within factors keyboard (visible, covered), action (EXE+S, EXE-S, and IMA), error type (higher-order planning, motor command), and source (reviewing the planning process, vision of the screen, vision of the keyboard, kinesthesis/touch, internal monitoring) was conducted on the number of reported errors. The results of the ANOVA are shown in Table 3. In the following, all the reported p-values and d-values refer to the post hoc comparisons.

*Comparisons between action conditions.* The significant main effect of action indicated that more errors were reported in EXE+S (*M* = 2.3 ± 0.3) than in EXE-S (*M* = 1.2 ± 0.2, *p* < 0.001, *d* = 0.93) and in IMA (*M* = 0.6 ± 0.2, *p* < 0.001, *d* = 1.52). The significant interaction between action and error type indicated that whereas the number of reported higher-order planning errors did not significantly differ between IMA and EXE-S, *p* = 0.69, *d* = 0.15, the number of reported motor command errors was significantly lower in IMA (*M* = 0.8 ± 0.2) than in EXE-S (*M* = 1.9 ± 0.3, *p* < 0.001, *d* = 0.96). However, the significant interaction between action, error type, and source indicated that the number of motor command errors did not significantly differ between IMA and EXE-S for errors detected by vision of the screen (*p* = 0.66, *d* = 0.41) and by vision of the keyboard (*p* = 0.23, *d* = 0.34).

*Comparisons between keyboard conditions.* The significant main effect of keyboard was modified by the significant interaction between keyboard and error type. Participants reported significantly more motor command errors with covered keyboard (*M* = 2.5 ± 0.5) than with visible keyboard (*M* = 1.6 ± 0.2, *p* = 0.032, *d* = 0.68). The number of reported higher-order planning errors did not significantly differ between keyboard conditions, *p* > 0.99, *d* = 0.

*Comparisons between error types.* The significant main effect of error type was modified by the significant interaction between error type and source. When participants detected errors by reviewing the planning process, they reported significantly more higher-order planning errors (*M* = 1 ± 0.3) than motor command errors (*M* = 0.3 ± 0.05, *p* = 0.014, *d* = 0.81). When participants detected errors by other sources, they reported significantly fewer higher-order planning errors than motor command errors, *p*_max_ = 0.02, *d*_min_ = 0.36. Further, the significant interaction between keyboard, action, and error type indicated that participants reported significantly fewer higher-order planning errors than motor command errors in all experimental conditions, *p*_min_ = 0.026, *d*_min_ = 0.56, except in IMA with covered keyboard, *p* = 0.36, *d* = 0.14.

*Comparisons between sources for error detection.* The significant main effect of source was modified by the significant interaction between action and source. In EXE+S, participants used vision of the screen (*M* = 5.3 ± 1.1) and kinesthesis/touch (*M* = 4.3 ± 0.5) significantly more often for error detection than the remaining sources (reviewing the planning process: *M* = 1 ± 0.2, vision of the keyboard: *M* = 0.03 ± 0.02, internal monitoring: *M* = 0.6 ± 0.2, *p*_max_ = 0.002, *d*_min_ = 2.39). In contrast, in EXE-S (*M* = 0.01 ± 0.01) and IMA (*M* = 0.2 ± 0.2), participants did not use vision of the screen significantly more often than any other source, *p*_min_ > 0.99, *d*_max_ = 0.03.

### 3.2. Both Typings Styles: Typing Style, Action, Error Type, and Source

An ANOVA with the between factor typing style (ten-finger typists, hunt-and-peck typists) and the within factors action (EXE+S, EXE-S, and IMA), error type (higher-order planning, motor command), and source (reviewing the planning process, vision of the screen, vision of the keyboard, kinesthesis/touch, internal monitoring) was conducted on the number of reported errors. The results of the ANOVA are shown in Table 4. In the following, all the reported p-values and d-values refer to the post hoc comparisons.

*Comparisons between action conditions.* Similar to the analysis of ten-finger typists, the significant main effect of action indicated that more errors were reported in EXE+S (*M* = 1.9 ± 0.2) than in EXE-S (*M* = 1.2 ± 0.1, *p* < 0.001, *d* = 0.93) and in IMA (*M* = 0.6 ± 0.1, *p* < 0.001, *d* = 0.86). The significant interaction between action and error type indicated that whereas the number of reported higher-order planning errors did not significantly differ between IMA (*M* = 0.5 ± 0.1) and EXE-S (*M* = 0.6 ± 0.1, *p* = 0.84, *d* = 0.14) significantly fewer motor command errors were reported in IMA (*M* = 0.6 ± 0.1) than in EXE-S (*M* = 1.7 ± 0.1, *p* < 0.001, *d* = 1.4). However, the significant interaction between typing style, action, error type, and source indicated that the difference between IMA and EXE-S was not significant for motor command errors detected by vision of the screen in both typing styles (*p*_min_ = 0.46, *d*_max_ = 0.54), by vision of the keyboard in ten-finger typists (*p* = 0.62, *d* = 0.26), and by reviewing the planning process in hunt-and-peck typists (*p* = 0.3, *d* = 0.46).

*Comparisons between typing styles.* The significant interaction between typing style, error type, and source indicated that hunt-and-peck typists detected significantly more higher-order planning errors by reviewing the planning process (*M* = 2.1 ± 0.2) than ten-finger typists (*M* = 1.1 ± 0.2, *p* = 0.002, *d* = 0.89). In contrast, hunt-and-peck typists detected significantly fewer higher-order planning errors by kinesthesis/touch (*M* = 0.7 ± 0.2) than ten-finger typists (*M* = 1.5 ± 0.2, *p* < 0.001, *d* = 0.73). Further, hunt-and-peck typists detected significantly more motor command errors by vision of the keyboard (*M* = 2.4 ± 0.3) than ten-finger typists (*M* = 0.6 ± 0.3, *p* < 0.001, *d* = 1.32).

*Comparisons between error types.* Similar to the analysis of ten-finger typists, the significant main effect of error type was modified by the significant interaction between error type and source. When participants detected errors by reviewing the planning process, they detected significantly more higher-order planning errors (*M* = 1.6 ± 0.2) than motor command errors (*M* = 0.3 ± 0.04, *p* < 0.001, *d* = 1.73). When participants detected errors by other sources, they detected significantly fewer higher-order planning errors than motor command errors, *p*_max_ = 0.001, *d*_min_ = 0.55. Further, the significant interaction between action and error type indicated that participants reported significantly fewer higher-order planning errors than motor command errors in in EXE+S (*p* < 0.001, *d* = 1.85) and EXE-S (*p* < 0.001, *d* = 1.6), but not in IMA, *p* = 0.93, *d* = 0.03.

*Comparisons between sources.* Similar to the analysis of ten-finger typists, the significant main effect of source was modified by the significant interaction between action and source. In EXE+S, participants used vision of the screen and kinesthesis/touch significantly more often for error detection than the remaining sources, *p*_max_ < 0.001, *d*_min_ = 1.02. In contrast, in EXE-S (*M* = 0.01 ± 0.01) and IMA (*M* = 0.2 ± 0.2), participants did not use vision of the screen significantly more often than any other source, *p*_min_ > 0.99, *d*_max_ = 0.06.

## 4. Discussion

In the present study, we investigated differences (and similarities) between MI and ME in error reports. To investigate the impact of typing style and the impact of feedback from the screen on differences between ME and MI, ten-finger typists and hunt-and-peck typists typed with vision of the screen, typed without vision of the screen, and typed in their imagination. To investigate the impact of feedback from the keyboard, ten-finger typists typed with visible and covered keyboard. Participants were asked to report what kind of error had occurred, and how they had detected the error.

### 4.1. The Role of Screen Visibility for Error Reports

In ME, we replicated the observation that more errors are reported with visual feedback from the screen than without visual feedback from the screen [25]. This underpins the importance of visual feedback from the screen and information about the action effects for explicit error detection and error reports in ME [30,31]. Since there is no actual feedback in MI, this indicates that the absence of actual distal action effects in MI is one major reason for differences between MI and ME [39]. To investigate MI, comparisons with ME conditions without distal action effects are therefore more appropriate.

In action conditions without screen visibility, we expected that typists may predict the action effects [32] and therefore imagine letters on the screen. Although the use of vision of the screen as a source for error detection did not significantly differ between EXE-S and IMA, it was in both conditions lower than in EXE+S. Indeed, vision of the screen was almost never used as a source for error detection in EXE-S and IMA. Visual feedback from distal action effects may not be used when the actual action effects are not available due to at least two reasons. First, distal action effects are not predicted by forward models [40], and therefore distal action effects are not imagined. Second, errors are predicted and imagined, but are primarily detected by other sources. For error detection in MI, proximal action effects (e.g., kinesthesis) may be used rather than distal action effects (e.g., on the screen) [30].

### 4.2. The Role of Keyboard Visibility

We observed more reported motor command errors with covered keyboard than with visible keyboard, whereas higher-order planning errors did not significantly differ between keyboard conditions. Thus, overall, more errors were reported (and committed, see analyses in the Supplement) with covered keyboard than with visible keyboard. Our results correspond with other studies showing that with covered keyboard, error rates increase either because typists are not able to identify the exact position of the target keys, are not able to optimally align their fingers on the keyboard [27], or because vision of the finger trajectories is needed for optimal performance [41]. In particular, our results indicate that visibility of the keyboard is an important source to prevent motor command errors. It is noteworthy that covering the keyboard increased the error rates, even in ten-finger typists [30]. Although ten-finger typists rarely use vision of the keyboard for error detection, they may sometimes need to look at keyboard and hands to identify the exact position of the target keys and to monitor the finger position.

Interestingly, the increase of reported motor command errors with covered keyboard was observed in MI in a similar way as in ME. Further, in MI and ME conditions with visible keyboard, participants reported using error detection by vision of the keyboard. In contrast, in MI and ME with covered keyboard, error detection by vision of the keyboard was not reported. This indicates that MI is influenced by task-related information from the environment in a similar way as ME. Most likely, vision of the target keys promotes the imagination of movements to them. The results are consistent with the assumption that the visibility of tools used in an action and the visibility of the action environment influences imagery quality positively [42].

### 4.3. Higher-Order Planning and Motor Command Errors

In line with our expectations [25], the number of reported higher-order planning errors did not significantly differ between IMA and EXE-S. We further observed that participants reported fewer motor command errors in IMA than in EXE-S. Motor command errors may not be reported in MI for several reasons. First, forward modeling may not occur in MI. If inhibition in MI prevents the generation of an efference copy, motor command errors cannot be predicted by forward models [24]. However, participants reported some motor command errors in MI, indicating that (at least sometimes) action effects are predicted in MI and that error monitoring occurs in MI [23]. Second, forward models may be imprecise. In line with this, in ten-finger typists, which may have more precise internal models than hunt-and-peck typists [14], motor command errors detected by vision of the keyboard did not significantly differ between IMA and EXE-S. In hunt-and-peck typists, motor command errors were reported less often in IMA than in EXE-S. Third, forward models may not always predict all action effects. Fourth, although forward models predict the action effects in MI, errors may not be reported in MI because internal error monitoring does not always occur. Deliberate MI requires attention to processes that are automatized in ME, which may leave fewer resources for monitoring motor command errors.

### 4.4. The Role of Typing Style

In contrast to our expectation that in MI, ten-finger typists may detect motor command errors better than hunt-and-peck typists [14], typing style alone (without taking sources for error detection into account) had no significant effect on the number of motor command or higher-order planning errors in ME and MI. The present results indicate that ten-finger typists may not have more precise internal models than hunt-and-peck typists [14]. Indeed, participants from both typing styles did not significantly differ in years of typing experience and hours they spend typing per week.

Although the number of reported errors (and committed errors, see Supplemental Material) did not significantly differ between typing styles, the typing styles differed in the use of sources for error detection. Ten-finger typists detected fewer motor command errors by vision of the keyboard than hunt-and-peck typists. This is in line with observations that error detection by vision of the keyboard is characteristic for hunt-and-peck typing [34,43]. Ten-finger typists detected more higher-order planning errors by kinesthesis/touch than hunt-and-peck typists. This is in line with observations that error detection by kinesthesis/touch is characteristic for ten-finger typing [34,43]. This typing style-specific use of sources for error detection did not significantly differ between MI and ME, indicating similar mechanisms.

We expected that more motor command errors are detected by internal monitoring in ten-finger typists than in hunt-and-peck typists [33], because the use of more fingers and the fixed finger-to-key assignment in ten-finger typists leads to more specific associations between letters and motor commands. However, this was not observed. In fact, our data showed that internal monitoring was not often reported as a source for error detection in both typing styles. The error signal from internal monitoring alone may not be strong enough to convince participants that an error actually occurred, and consequently might not always result in an error report. This may be the case for error signals from other sources for error detection as well, but may particularly apply to internal monitoring. Another explanation for the diverging results between the present and the previous study [34] is that different methods were used to asses internal monitoring. When asked to rate the sources for error detection, ten-finger typists may report that they sometimes know about an error without being able to name a specific source because they may usually not pay much attention to how they detect errors. However, in the present study, participants were explicitly asked to report how they detected every single error, which may have promoted conscious error monitoring.

### 4.5. Limitations and Perspectives

In the present study, one source for error detection was assigned to each error. However, it is possible that during error detection, information from different sources is combined, and an error is detected if the combined information reaches a certain threshold.

Not all errors can be assigned to higher-order planning errors and motor command errors with utter certainty. For instance, in difficult words, it might be unclear whether an omission of a letter that has to be typed twice in a row has occurred because the participant does not know the right spelling (the error would then be classified as a higher-order planning error, because the spelling was correct on the template) or due to a failure to create a sufficiently strong motor command (the error would then be classified as a motor command error). Hence, error subcategories that could not be unequivocally assigned to our categories were not included in the analysis. Still, there might be a few errors within the subcategories included in the analysis, in which the assignment to higher-order planning or motor command errors might be debated. On rare occasions, errors classified as motor command errors were detected by reviewing the planning process, which does not seem plausible. 

It also has to be noted that different authors provide different reasons for the occurrence of some errors [28,29,44]. The classification that we adopted was based on the literature on copy typing [28,29]. In spoken language, similar errors may occur for different reasons [44]. For example, an interchange error may occur due to permutation of the motor commands [29] or due to phonological similarity of two phonemes [44]. We cannot rule out the possibility that covert speech contributed to some typing errors.

Some of the error types and sources for error detection in the present study were characteristic for copy typing. For instance, word substitutions may not or to a lesser degree occur in free writing or typing from dictation. Future studies may examine error detection in MI using other tasks in which other error types are observed more commonly, and that require the sources for error detection to different degrees.

From a methodological viewpoint for imagery research, this study contributes to emerging evidence that indicates when MI and ME are compared, it is important to have a condition in which ME is conducted without distal action effects [12,32,40]. An ME condition without distal action effects enables one to distinguish which differences between MI and ME are due to the lack of distal action effects and which differences are due to other mechanisms.

An application of MI research is mental practice. Mental practice refers to the repeated use of MI to enhance motor performance [45]. The observation that fewer motor command errors were reported in IMA than in EXE-S and that some errors are therefore presumably not detected in MI could explain why mental practice is less effective than physical practice [45]. Error detection and error correction play a crucial role in motor learning [17]. Learning due to an optimization of internal models may be weaker in mental practice than in physical practice because fewer error signals occur.

## 5. Conclusions

In MI, not all sources that provide information for error detection in ME are used. For instance, in copy typing, vision of the screen is not used for error detection in MI. Distal action effects may not be predicted or not be monitored in MI. Further, the probability of detecting errors in MI depends on whether errors occur during higher-order planning or during the specification of the motor commands. Fewer motor command errors are reported in MI than in ME. This can partly, but not completely, be explained by the lack of distal action effects in MI. The results underpin the importance of visual feedback from the screen for error detection in copy typing but also show that other sources for error detection are used, too. In both MI and ME, typing style did not influence the type and number of reported errors, but influenced how errors are detected. We assume that forward modeling does occur in MI. However, less attention may be paid to error monitoring in MI, because attention is required for other processes that are automatized in ME but not in MI.

## Figures and Tables

**Table 1:** Demographic data and data related to typing experience. The typing test consisted of 670 characters including spaces.

	Ten-Finger Typists		Hunt-and-Peck Typists		*t* (52)	*p*
Sex (female/male), *N*	21/4		25/4			
Handedness (left/right), *N*	3/22		3/26			
Age in years, *M* (*SD*)	25	(6.6)	24	(6.2)	0.6	0.54
Typing experience in years, *M* (*SD*)	12.1	(5.7)	12	(3)	<0.1	0.97
Hours spend typing per week, *M* (*SD*)	9.2	(8.5)	10.4	(9.8)	0.5	0.64
Typing test: Correct keystrokes/minute, *M* (SD)	246	(48)	178	(36)	6.6	<0.001
Typing test: % incorrect characters, *M* (*SD*)	0.026	(0.009)	0.026	(0.012)	0.1	0.9

**Table 2:** Means and standard errors (±) of the number of reported errors depending on typing style (ten-finger typists and hunt-and-peck typists), action condition (execution with visible screen [EXE+S], execution with covered screen [EXE-S], and imagination [IMA]), keyboard (visible and covered), error type (higher-order planning and motor command), and error detection source (reviewing the planning process, vision of the screen, vision of the keyboard, kinesthesis/touch, and internal monitoring). Note that hunt-and-peck typists did not perform the conditions in which the keyboard was covered.

	Ten-Finger Typists (*N* = 25)						Hunt-and-Peck Typists (*N* = 29)		
	Visible Keyboard			Covered Keyboard			Visible Keyboard		
	EXE+S	EXE-S	IMA	EXE+S	EXE-S	IMA	EXE+S	EXE-S	IMA
**Higher-Order Planning Errors**											
reviewing the planning	1.7	0.9	0.6	1.5	0.9	0.4	2.3	2.1	1.8
	±0.4	±0.3	±0.3	±0.5	±0.3	±0.2	±0.3	±0.3	±0.3
vision of the screen	1.6	0.0	0.0	1.8	0.0	0.1	1.2	0.0	0.0
	±0.3	±0.0	±0.0	±0.4	±0.0	±0.1	±0.3	±0.0	±0.0
vision of the keyboard	0.0	0.1	0.2	0.0	0.0	0.0	0.1	0.4	0.4
	±0.1	±0.1	±0.2	±0.0	±0.0	±0.0	±0.1	±0.1	±0.1
kinesthesis/touch	1.6	1.3	1.5	1.4	1.8	1.6	0.6	0.9	0.7
	±0.2	±0.2	±0.4	±0.3	±0.3	±0.6	±0.2	±0.2	±0.4
internal monitoring	0.2	0.2	0.2	0.4	0.4	0.0	0.1	0.2	0.1
	±0.1	±0.1	±0.1	±0.2	±0.1	±0.0	±0.1	±0.1	±0.1
sum of all sources	5.2	2.6	2.5	5.1	3.1	2.0	4.3	3.6	3.0
	±0.6	±0.5	±0.7	±0.9	±0.5	±0.7	±0.5	±0.4	±0.5
**Motor Command Errors**											
reviewing the planning	0.5	0.4	0.0	0.4	0.1	0.2	0.2	0.2	0.4
	±0.1	±0.1	±0.1	±0.2	±0.1	±0.1	±0.1	±0.1	±0.1
vision of the screen	5.8	0.0	0.2	11.9	0.0	0.6	4.1	0.0	0.1
	±1.0	±0.0	±0.1	±3.1	±0.0	±0.6	±0.9	±0.0	±0.1
vision of the keyboard	0.1	1.0	0.5	0.0	0.0	0.0	2.5	4.1	0.7
	±0.4	±0.5	±0.3	±0.0	±0.0	±0.0	±0.4	±0.5	±0.2
kinesthesis/touch	6.5	5.9	2.3	7.8	9.1	3.4	7.1	4.5	1.2
	±1.0	±0.8	±0.6	±1.1	±2.1	±1.0	±0.9	±0.7	±0.5
internal monitoring	0.4	0.6	0.1	1.3	1.9	0.7	0.6	0.6	0.1
	±0.2	±0.2	±0.1	±0.4	±0.5	±0.4	±0.2	±0.2	±0.1
sum of all sources	13.3	8.0	3.1	21.4	11.1	5.0	14.5	9.4	2.5
	±1.4	±0.9	±0.9	±3.7	±2.4	±1.5	±1.3	±0.9	±0.7

**Table 3:** Statistical values of the ANOVA on the number of reported errors. Factors and factor levels were action (execution with visible screen, execution with covered screen, imagination), error type (higher-order planning, motor command), source (reviewing the planning process, vision of the screen, vision of the keyboard, kinesthesis/touch, internal monitoring), and keyboard (visible and covered).

	*F*	*df1, df2*	*p*	η*²_p_*
Keyboard	5.3	1, 24	0.03	0.18
Action	44.3	1.5, 35.2	<0.001	0.65
error type	36.1	1, 24	<0.001	0.60
Source	36.7	2.2, 51.7	<0.001	0.61
keyboard × action	4.0	1.3, 31.4	0.045	0.14
keyboard × error type	4.9	1, 24	0.037	0.17
keyboard × source	3.6	1.9, 45.4	0.039	0.13
action × error type	37.8	1.8, 42.1	<0.001	0.61
action × source	15.4	2, 47.9	<0.001	0.39
error type × source	24.6	1.7, 40	<0.001	0.51
keyboard × action × error type	7.1	1.6, 37.2	0.005	0.23
keyboard × action × source	4.1	1.4, 34.2	0.037	0.15
action × error type × source	14.8	1.8, 42.8	<0.001	0.38
keyboard × action × error type × source	3.0	1.5, 34.7	0.08	0.11

**Table 4:** Statistical values of the ANOVAs on the number of reported errors. Factors and factor levels were action (execution with visible screen, execution with covered screen, imagination), error type (higher-order planning, motor command), source (reviewing the planning process, vision of the screen, vision of the keyboard, kinesthesis/touch, internal monitoring), and typing style (ten-finger typists and hunt-and-peck typists).

	*F*	*df1, df2*	*p*	η*²_p_*
typing style	0.3	1, 52	0.56	0.01
action	100.7	1.8, 91.5	<0.001	0.66
error type	119.0	1, 52	<0.001	0.7
source	49.1	2.1, 107.2	<0.001	0.49
typing style × action	1.1	1.8, 91.5	0.33	0.02
typing style × error type	0.2	1, 52	0.64	<0.01
typing style × source	6.0	2.1, 107.2	0.003	0.1
action × error type	63.2	2, 104	<0.001	0.55
action × source	25.3	3.6, 185.2	<0.001	0.33
error type × source	62.7	2.5, 131.2	<0.001	0.55
typing style × action × error type	1.9	2, 104	0.16	0.04
typing style × action × source	2.4	3.6, 185.2	0.062	0.04
action × error type × source	21.7	2.5, 131.2	<0.001	0.3
typing style × action × error type × source	3.0	4.7, 242.2	0.013	0.06

## Supplementary Materials

Additional analyses are available online at https://www.mdpi.com/2411-5150/3/4/66/s1. The data are available online at https://doi.org/10.17605/OSF.IO/82TG3.

## Appendix A

Appendix A gives an overview of all the error categories in copy typing (Table A1). For each error category, an example is provided.

**Table A1:** Description and frequencies (sum over participants) of reported errors separately for the different error types and their subcategories. The templates included 411 words/2741 keystrokes.

	Frequency	Template	Error Examples
**higher-order planning errors**	741		
insertion word	47	Oktoberfest	Oktoberpartyfest
insertion space key/line break	35	Oktoberfest	Oktober fest
insertion comma/full stop	19	Oktoberfest	Oktoberfest,
insertion grammar (i.e., plural)	56	wichtige	wichtigste
substitution word	134	Oktoberfest	Maiparty
omission line	23	viele Worte	
omission word	59	Oktoberfest	das Fest
omission grammar/plural	44	Personen	Person
omission comma/full stop	89	Berg.	Berg
omission space/line break	55	Oktoberfest	dasOktoberfest
capitalization	60	sondern	Sondern
no capitalization	120	Oktoberfest	oktoberfest
**motor command errors**	1985		
insertion double	71	Oktoberfest	Oktoberffest
insertion homologous key	23	Innsbruck	Iennsbruck
insertion neighbor key	377	Oktoberfest	Oktoberfgest
insertion other key	64	Oktoberfest	Oktoberfoest
substitution homologous key	68	Oktoberfest	Oktoberjest
substitution neighbor key	711	Oktoberfest	Oktobergest
substitution other key	197	Oktoberfest	Oktoberkest
transposition homologous keys	14	Haustier	Hausteir
transposition other keys	247	Oktoberfest	Oktoberefst
interchange	3	Oktoberfest	Oktobfreest
migration	34	Oktoberfest	Oktobertfes
alternation	39	dieses	disese
doubling	25	Innsbruck	Inssbruck
capitalization second letter	112	Oktoberfest	OKtoberfest
**other errors**	557		
finger positions	6	Oktoberfest	Oktobergrdz
insertion shift	63	Oktoberfest	#Oktoberfest
phonological substitutions	86	Oktoberfest	Oktoberfesd
omission double	31	Innsbruck	Insbruck
omission other key	371	Oktoberfest	Oktoberest

## Appendix B

Appendix B gives an overview of the sources for error detection in copy typing (Table A2).

**Table A2:** List of sources for error detection and their subcategories. Denoted is the frequency (sum over participants) with which each source was mentioned (# reported errors) and the percentage of each source of reported errors (%).

	# Reported Errors	%
**review of the planning process**	381	11.7
forgetting to type	45	1.4
misreading of the template	320	9.8
orthographic mistake	16	0.5
**vision of the screen**	704	21.6
**vision of the keyboard and fingers**	343	10.5
**feeling of the keyboard or fingers (kinesthesis/touch)**	1613	49.6
Misstroke	381	11.7
wrong key	461	14.2
wrong finger	158	4.9
wrong movement	613	18.8
**internal monitoring**	211	6.5
