# Supplementary material for: Errors in Imagined and Executed Typing
Source: Vision (Basel). 2019 Nov 20;3(4):66. doi: 10.3390/vision3040066 (PMC6969896; doi:10.3390/vision3040066)
Supplement: Supplementary file 1 [file vision-03-00066-s001.pdf]

SUPPLEMENTAL MATERIAL

Errors in imagined and executed typing

Stephan F. Dahm [1] & Martina Rieger [1]
[1] UMIT - University for Health Sciences Medical Informatics and Technology

Overview

This document includes additional analyses that may be of interest to some readers. In particular, we report a more detailed analysis of the data of the execution conditions here, as this would have been beyond the scope of the manuscript. First, we analyzed the number of actual errors. Second, we analyzed the hit rate (percentage of reported errors of actual errors). Third, we analyzed the false alarm rate (participants reported an error but had typed correctly). Fourth, we analyzed the percentage of actual errors of reported errors. Fifth, we analyzed the percentage of incorrectly identified errors (participants correctly reported that an error had occurred, but were not correct about what had actually gone wrong). We also report one analysis here in which imagination and execution are compared. As the sixth dependent variable, we analyzed the position of reported errors within words (beginning, middle, or end of words).

1. Number of actual errors in the execution conditions

To investigate the effects of our independent variables on the actual occurrence of higher-order-planning and motor command errors, we analyzed the number of actual errors (reported and unreported errors). Here, we categorized higher-order-planning and motor command errors based on what typists actually did wrong and not on error reports, as in the manuscript. Means and standard errors of the number of actual errors are shown in Figure SM1.

To analyze the effects of covering the keyboard in ten-finger typists, an ANOVA with the within factors keyboard (visible, covered), screen (visible, covered), and error type (higher-order-planning, motor command) was conducted. The significant main effect of error type, $F(1,24) = 78.1$, $p < .001$, $\eta^2_p = .77$, indicated significantly more committed motor command errors ($M = 24.4 \pm 2.5$) than higher-order-planning errors ($M = 7.5 \pm 0.7$). The significant main effect of keyboard, $F(1,24) = 12.1$, $p = .002$, $\eta^2_p = .33$, was modified by the significant interaction between keyboard and error type, $F(1,24) = 11.8$, $p = .002$, $\eta^2_p = .33$. Significantly more motor command errors were committed with covered keyboard ($M = 31 \pm 4.2$) than with visible keyboard ($M = 17.7 \pm 1.4$, $p = .002$). The number of committed higher-order-planning errors did not significantly differ between keyboard conditions ($M = 7.9 \pm 0.9$, $M = 7 \pm 0.6$, $p = .161$). The significant interaction between keyboard and screen, $F(1,24) = 5.8$, $p = .024$, $\eta^2_p = .19$, indicated that the difference between covered and visible keyboard was significantly larger with covered screen ($M_{Diff} = 8.6 \pm 2.4$) than with visible screen ($M_{Diff} = 5.6 \pm 1.8$ $p = .024$). The remaining effects were not significant, screen: $F < 1$; screen x error type: $F(1,24) = 2.5$, $p = .127$, $\eta^2_p = .09$; keyboard x screen x error type: $F = 1$.

To compare the two typing styles, an ANOVA with the between factor typing style (ten-finger typists, hunt-and-peck typists) and the within factors screen (visible, covered) and error type (higher-order-planning, motor command) was conducted. The significant main effect error type, $F(1,52) = 135.5$, $p < .001$, $\eta^2_p = .72$, indicated significantly more committed motor command errors ($M = 17.7 \pm 1.1$) than higher-order-planning errors ($M = 7.8 \pm 0.5$). The remaining effects were not significant, typing style: $F < 1$; screen, $F(1,52) = 3.7$, $p = .061$, $\eta^2_p = .07$, typing style x screen: $F = 1$; screen x error type: $F < 1$; typing style x screen x error type: $F(1,52) = 1.4$, $p = .24$, $\eta^2_p = .03$.

In sum, in copy typing, motor command errors occur more frequently than higher-order-planning errors. The number of committed motor command errors is higher with covered keyboard than with visible keyboard, whereas the number of committed higher-order-planning errors does not

significantly differ between keyboard conditions. These results are consistent with the analysis of reported errors in the manuscript. In the execution conditions, the number of reported motor command errors was higher than the number of higher-order-planning errors and keyboard visibility had a similar influence on error reports. Importantly, this indicates that reported errors can serve as a reasonable approximation of actual errors, as they behave similarly under different conditions.

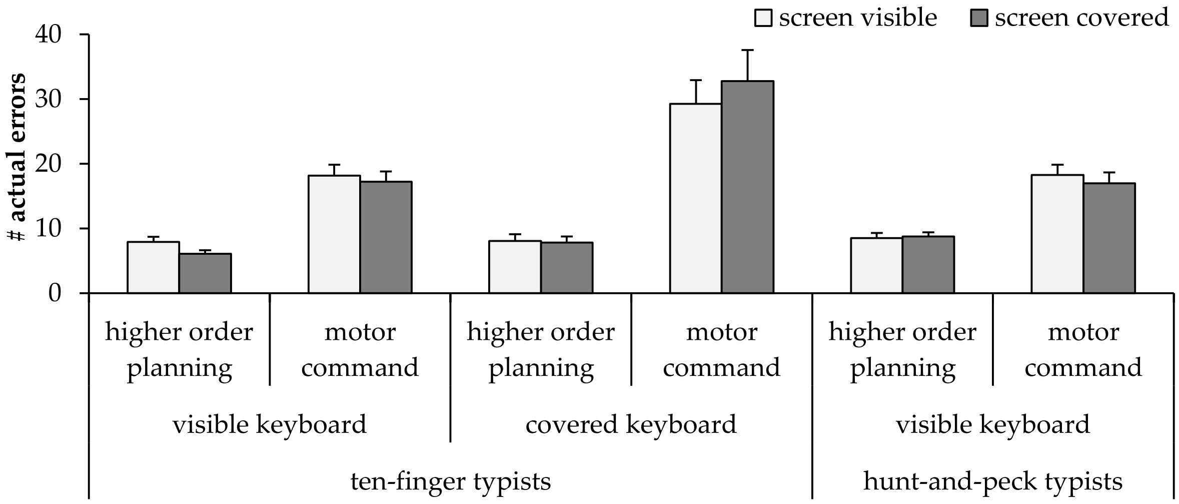

Figure SM1. Means and standard errors of the number of actual higher-order-planning and motor command errors in ten-finger typists and hunt-and-peck typists with visible and covered screen. In ten-finger typists the keyboard was either visible or covered.

2. Hit rate (percentage of reported errors of actual errors) in the execution conditions

To investigate the effects of our independent variables on error reports, we analyzed the percentage of reported errors of actual errors (hit rate). We used the percentage because the number of reported errors depends on the number of actual errors and we wanted to control for differences in the number of actual errors between conditions. Means and standard errors of the percentage of reported errors of actual errors are shown in Figure SM2.

To analyze the effects of covering the keyboard in ten-finger typists, an ANOVA with the within factors keyboard (visible, covered), screen (visible, covered), and error type (higher-order-planning, motor command) was conducted. The significant main effect screen, $F(1,24) = 102.3$, $p < .001$, $\eta^2_P = .81$, indicated a significantly higher hit rate with visible screen ($M = 65.3 \pm 2.9\%$) than with covered screen ($M = 34.7 \pm 2.6\%$). The significant interaction between screen and error type, $F(1,24) = 7.3$, $p < .001$, $\eta^2_P = .23$, indicated a significantly lower hit rate in higher-order-planning errors than in motor command errors with visible screen ($p = .026$), but not with covered screen ($p = .154$). The remaining effects were not significant (error type: $F < 1$, keyboard: $F(1,24) = 2.8$, $p = .11$, $\eta^2_P = .1$, error type x keyboard: $F < 1$, screen x keyboard: $F < 1$, error type x screen x keyboard: $F(1,24) = 3.7$, $p = .067$, $\eta^2_P = .13$). A closer look at the conditions with covered screen (dark bars) in Figure SM2, seemed to indicate the hit rate was lower in motor command errors with covered keyboard than in the other conditions. We therefore decided to perform posthoc analyses of the three-way interaction even though it did not reach significance in the ANOVA. The posthoc analysis indicated a significantly lower hit rate in motor command errors with covered keyboard than in motor command errors with visible keyboard when the screen was covered ($p = .003$), but not when the screen was visible ($p = .74$). This was not the case in higher-order-planning errors ($p_{min} = .32$).

To compare the two typing styles, an ANOVA with the between factor typing style (ten-finger typists, hunt-and-peck typists) and the within factors screen (visible, covered) and error type (higher-order-planning, motor command) was conducted. Again, the significant main effect screen, $F(1,52) = 91.1$, $p < .001$, $\eta^2_P = .64$, indicated a significantly higher hit rate with visible screen ($M = 64.4 \pm 2.1\%$) than

with covered screen (M = 41.4±2.1%). The significant interaction between typing style and screen, $F(1,52) = 5.9$, $p = .019$, $\eta^2_p = .1$, indicated that the difference between visible and covered screen was significantly larger in ten-finger typists than in hunt-and-peck typists ($p = .019$). The significant main effect error type, $F(1,52) = 16.5$, $p < .001$, $\eta^2_p = .24$, was modified by the significant interaction between error type and typing style, $F(1,52) = 7.5$, $p = .008$, $\eta^2_p = .13$, indicating a significantly lower hit rate in higher-order-planning than in motor command errors in hunt-and-peck typists ($p < .001$), but not in ten-finger typists ($p = .37$). The remaining effects were not significant, typing style: $F < 1$, screen x error type: $F(1,52) = 1.5$, $p = .23$, $\eta^2_p = .03$, typing style x screen x error type: $F < 1$.

In sum, the hit rate was higher with visible screen than with covered screen. This is consistent with our interpretation in the manuscript that visual feedback from the screen is important to detect and report errors. The hit rate for motor command errors was higher than the hit rate for higher-order-planning errors in nearly all conditions. Only when both screen and keyboard were covered, the reverse was the case (lower hit rate in motor command errors than in higher-order-planning errors). Further, when both screen and keyboard are covered the hit rate in motor command errors is lower than with covered screen and visible keyboard. This is consistent with our interpretation with the manuscript that when the screen is not available, the keyboard may serve as a secondary source of feedback to detect and report errors. In line with findings of the manuscript, we observed that visibility of the screen is more important in ten-finger typists than in hunt-and-peck typists for error detection.

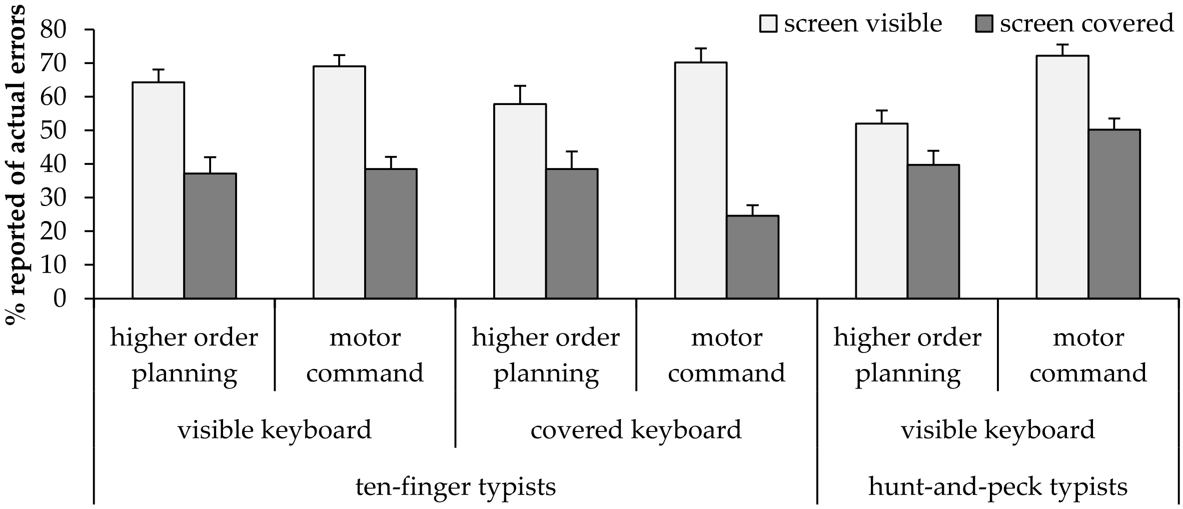

Figure SM2. Means and standard errors of the percentage of reported errors of actual errors in ten-finger typists and hunt-and-peck typists with visible and covered screen. In ten-finger typists the keyboard was either visible or covered.

3. False alarm rate in the execution conditions

An error report can occur in two instances: either an error had actually occurred (hit) or an error did not occur but was reported (false alarm). Here, we analyze the false alarm rate in the execution conditions: false alarms / correct keystrokes * 100. Means and standard errors of the false alarm rate are shown in Figure SM3.

To analyze the effects of covering the keyboard in ten-finger typists, an ANOVA with the within factors keyboard (visible, covered) and screen (visible, covered) was conducted. The significant main effect of screen, $F(1,24) = 14.6$, $p = .001$, $\eta^2_p = .38$, indicated a significantly higher false alarm rate with covered screen (M = 0.1±0.02%) than with visible screen (M =0.033±0.005%). The main effect keyboard, $F(1,24) < 1$, and the interaction between screen and keyboard, $F < 1$, were not significant.

To compare the two typing styles, an ANOVA with the between factor typing style (ten-finger typists, hunt-and-peck typists) and the within factor screen (visible, covered) was conducted. The

significant main effect of screen, $F(1,52) = 10.5$, $p = .002$, $\eta^2_p = .17$, indicated a significantly higher false alarm rate with covered screen (M = 0.082±0.01%) than with visible screen (M = 0.048±0.007%). The interaction between screen and typing style, $F(1,52) = 3.2$, $p = .079$, $\eta^2_p = .06$, and the main effect typing style, $F < 1$, were not significant.

In sum, the false alarm rate is higher with covered screen than with visible screen. This result indicates that screen visibility is not only important for error detection, but also for knowing that one typed correctly.

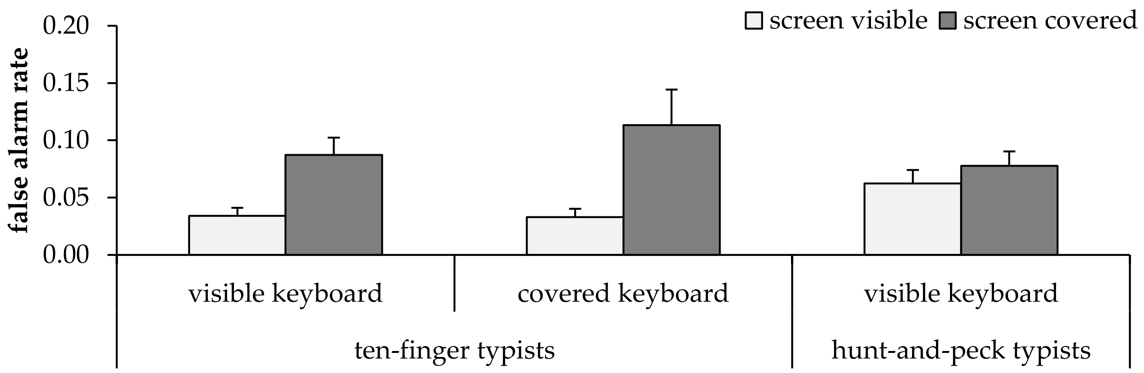

Figure SM3. Means and standard errors of the false alarm rate in execution.

4. Percentage of actual errors of reported errors in the execution conditions

An error report can occur in two instances: either an error had actually occurred (hit) or an error did not occur but was reported (false alarm). In imagination, we cannot distinguish between hits and false alarms. Hence, reported errors include false alarms in the manuscript. Here, we analyze the percentage of actual errors of reported errors: (reported errors - false alarms) / reported errors * 100. Means and standard errors of the percentage of actual errors of reported errors are shown in Figure SM4.

To analyze the effects of covering the keyboard in ten-finger typists, an ANOVA with the within factors keyboard (visible, covered) and screen (visible, covered) was conducted. The significant main effect of screen, $F(1,24) = 58.2$, $p < .001$, $\eta^2_p = .71$, indicated a significantly higher percentage of actual errors of reported errors with visible screen (M = 96.2±0.6%) than with covered screen (M = 84.5±1.6%). The main effect keyboard, $F(1,24) = 2.6$, $p = .12$, $\eta^2_p = .1$, and the interaction between screen and keyboard, $F < 1$, were not significant.

To compare the two typing styles, an ANOVA with the between factor typing style (ten-finger typists, hunt-and-peck typists) and the within factor screen (visible, covered) was conducted. The significant main effect of screen, $F(1,52) = 27$, $p < .001$, $\eta^2_p = .34$, indicated a significantly higher percentage of actual errors of reported errors with visible screen (M = 95±0.9%) than with covered screen (M = 84.6±1.6%). The significant interaction between screen and typing style, $F(1,52) = 5.2$, $p = .026$, $\eta^2_p = .09$, indicated that the difference between screen conditions was significantly larger in ten-finger typists than in hunt-and-peck typists ($p = .026$). The main effect typing style was not significant, $F < 1$.

In sum, the percentage of actual errors of reported errors is higher with visible screen than with covered screen. This is even more prominent in ten-finger typists than in hunt-and-peck typists indicating the importance of the screen for error detection in ten-finger typists. The findings go in line with lower false alarm rates and higher hit rates with visible screen than with covered screen. Again, this underpins the importance of the screen in copy typing to detect and report errors and to know that one typed correctly.

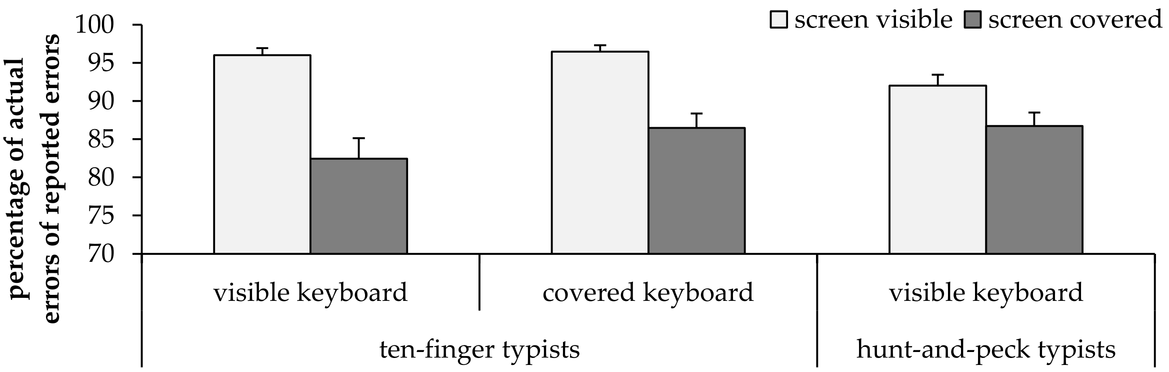

Figure SM4. Means and standard errors of the percentage of actual errors of reported errors in execution.

5. Percentage of incorrectly identified errors in the execution conditions

Error reports were conducted in two steps. In the first step, participants reported that an error occurred, in the second step they reported what had gone wrong. It can happen that participants correctly reported that an error occurred, but were not correct about what had actually gone wrong. Those errors are incorrectly identified errors. Again, we cannot distinguish between correctly and incorrectly identified errors in imagination. Therefore, incorrectly identified errors are included in reported errors in the manuscript. Here we analyze the percentage of incorrectly identified errors: incorrectly identified errors / reported errors * 100. Means and standard errors of the percentage of incorrectly identified errors of reported errors are shown in Figure SM5.

To analyze the effects of covering the keyboard in ten-finger typists, an ANOVA with the within factors keyboard (visible, covered) and screen (visible, covered) was conducted. The significant main effect of screen, $F(1,24) = 22.7$, $p < .001$, $\eta^2_p = .49$, indicated significantly more incorrectly identified errors with covered screen ($M = 8.1 \pm 1.4\%$) than with visible screen ($M = 1.2 \pm 0.3\%$). The main effect keyboard, $F(1,24) = 2$, $p = .171$, $\eta^2_p = .08$, and the interaction between screen and keyboard, $F < 1$, were not significant.

To compare the two typing styles, an ANOVA with the between factor typing style (ten-finger typists, hunt-and-peck typists) and the within factor screen (visible, covered) was conducted. The significant main effect of screen, $F(1,52) = 45.4$, $p < .001$, $\eta^2_p = .47$, indicated significantly more incorrectly identified errors with covered screen ($M = 7.3 \pm 0.8\%$) than with visible screen ($M = 1.5 \pm 0.4\%$). The main effect typing style, $F < 1$, and the interaction between screen and keyboard, $F < 1$, were not significant.

In sum, the percentage of incorrectly identified errors was higher with covered screen than with visible screen. Keyboard visibility and typing style had no significant influence on the percentage of incorrectly identified errors. Again, this underpins the importance of the screen in copy typing for knowing what one has actually done.

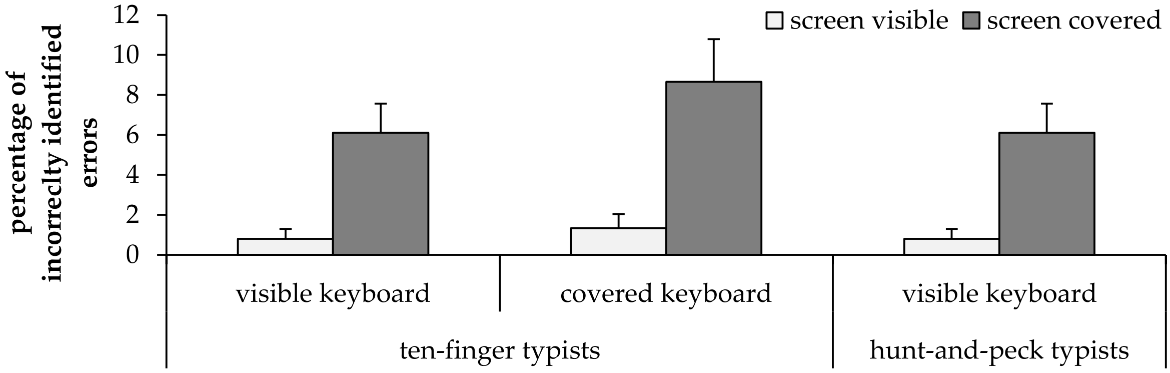

Figure SM5. Means and standard errors of the percentage of incorrectly identified errors of reported errors in execution.

6. Error position in execution and imagination

One might argue, that participants might have waited to report errors until the end of a word (even though they were instructed to stop typing and report errors immediately) and then forgot to report errors that occurred at the beginning of words. To explore whether errors that occur at different positions within words were reported to a different extent, we analyzed at what position in a word (beginning, middle, or end) reported errors occurred. If the word length was a multiple of 3, the word was divided into three equal sections. If the word length was 1 more than a multiple of 3, the middle section had one additional letter. If the word length was 1 less than a multiple of 3, the middle section had one letter less. Means and standard errors of the number of reported errors, depending on error position, are shown in Figure SM6. In the following analyses, we focus only on effects in which the factor position played a role.

To analyze the effects of covering the keyboard in ten-finger typists, an ANOVA with the within factors keyboard (visible, covered), position (beginning, middle, end), and action (EXE+S, EXE-S, IMA) was conducted. The significant main effect of position, $F(1.6,38.3) = 26.8$, $p < .001$, $\eta^2_p = .53$, was modified by the significant interaction between position and action, $F(4,96) = 9.3$, $p < .001$, $\eta^2_p = .28$. The interaction indicated significantly more reported errors at the beginning of words than at the middle and end of words in EXE+S ($p_{max} < .001$) and EXE-S ($p_{max} = .001$), but not in IMA ($p_{min} = .13$). The significant interaction between position and keyboard, $F(2,47.2) = 3.8$, $p = .029$, $\eta^2_p = .14$ indicated significantly more reported errors with covered keyboard ($M = 6.8 \pm 1$) than with visible keyboard ($M = 4.7 \pm 0.6$) at the beginning of words ($p = .018$), but not at the middle ($p = .051$) or end of words ($p = .059$). The three way interaction (position x action x keyboard) was not significant, $F < 1$.

To compare the two typing styles, an ANOVA with the between factor typing style (ten-finger typists, hunt-and-peck typists) and the within factors position (beginning, middle, end), and action (EXE+S, EXE-S, IMA) was conducted. The significant main effect of position, $F(1.8,95.3) = 18.8$, $p < .001$, $\eta^2_p = .27$, was modified by the significant interaction between position and action, $F(4,208) = 6.5$, $p < .001$, $\eta^2_p = .11$. In EXE+S, significantly more errors were reported at the beginning of words than at the middle and end of words, $p_{max} < .001$. In EXE-S, significantly more errors were reported at the beginning of words than at the middle of words, $p = .001$. In IMA, the number of reported errors did not significantly differ between positions, $p_{min} = .76$. The interaction between typing style and position, $F(1.8,95.3) = 2.6$, $p = .082$, $\eta^2_p = .05$, and the interaction between typing style, action, and position, $F(4,208) = 2.4$, $p = .054$, $\eta^2_p = .04$, were not significant.

In sum, in execution, more error reports referred to errors at the beginning of a word than to errors at the end of a word. In imagination, the number of reported errors does not significantly differ between positions within words. Further, more errors were reported with covered keyboard than with visible keyboard, but only when the error report referred to errors at the beginning of words. Overall, there is no indication in our data that participants may have forgotten to report errors at the beginning of a word.

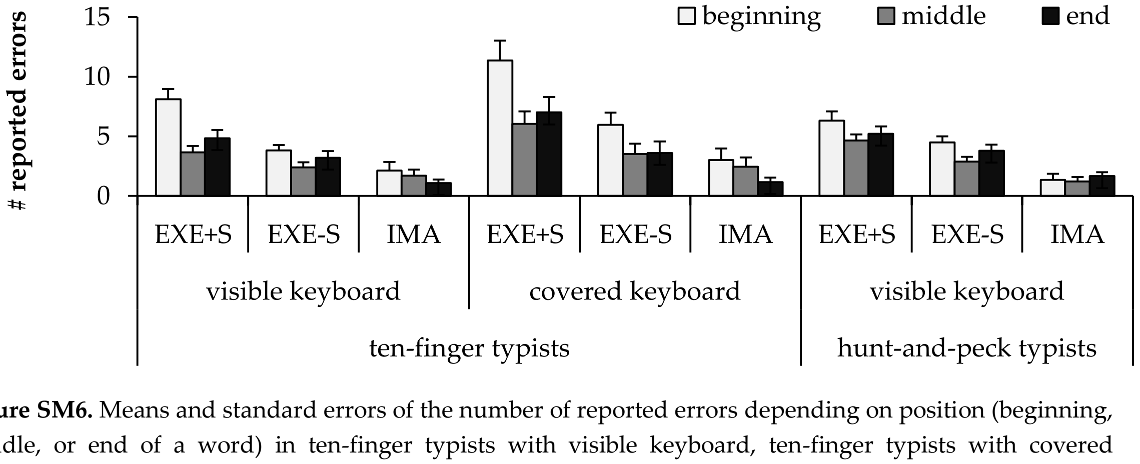

Figure SM6. Means and standard errors of the number of reported errors depending on position (beginning, middle, or end of a word) in ten-finger typists with visible keyboard, ten-finger typists with covered keyboard and hunt-and-peck typists with visible keyboard. The action conditions were execution with visible screen (EXE+S), execution with covered screen (EXE-S) and imagination (IMA).
